# On the Nature of the Waxy, Lardaceous, or Amyloid Deposit

**Published:** 1868-12

**Authors:** William H. Dickinson


### ON THE NATURE OF THE WAXY, LARDACEOUS, OR AMYLOID DEPOSIT.

By WILLIAM H. DICKINSON, M.D., etc.

The author has had the opportunity of observing 60 cases of waxy or amyloid degeneration of the solid organs, and has found that in 46 of these cases there was a well-attested history of suppuration, and that in 4 others suppuration had probably preceded the outbreak of the disease. Comparing this result with those shown by the cases collected by Dr. Wilks (*Guy's Hospital Reports*, 1856 and 1862) and by Dr. Stewart (*Edinburgh Monthly*, 1861 and 1864, and *Brit. and For. Med.-Chir. Rev.*, 1866), he finds that of 104 cases, suppuration had existed in 83; and the fact that these two gentlemen collected their cases without reference to antecedent suppuration, makes these figures more valuable.

In speaking of the test for this kind of degeneration, iodine and sulphuric acid, he says that he has never been able to produce the blue tint spoken of by Virchow, but that a reddish-brown color followed their application to the diseased tissue, instead of the yellow color which is obtained normally. He has found, moreover, that we possess in the sulphate of indigo as good a test as iodine; the healthy liver, when soaked in a weak solution of this salt, becomes of a blue color, which changes rapidly to green, but a waxy liver so treated retains the color. The colors obtained by iodine and sulphate of indigo are not destroyed if the affected tissue be soaked in alcohol, acids, or aqua ammoniæ, but fade when it is treated with a solution of caustic soda or potassa; and a waxy liver first treated with a solution of either of these alkalies, fails to respond to either test. Dr. Dickinson naturally infers from this, that there is a deficiency of these substances in the organs which have undergone this form of degeneration, and this inference he proves to be correct by a comparison of the analysis of a healthy liver with that of a waxy liver; in the latter soda and potassa are found to be decidedly diminished in quantity, and this deficiency he explains by the suppuration. Pus, as is well known, contains both these substances in larger proportion than the blood; hence, a drain of this kind cannot long continue without the occurrence of the result above indicated. He thinks that the deposit in the organs consists essentially of dealkalized fibrin, and says that if fibrin be treated with dilute hydrochloric acid, and then the solution evaporated to dryness, a substance will be obtained which reacts with iodine precisely as the amyloid liver. He proposes to apply the term "Depurative" to the disease, as significant of the process which is its most frequent cause. The objection to the word is, as he himself says, its frequent use in another sense.

The practical deduction to be drawn from this paper is, that the exhibition of alkalies is imperatively called for, not merely during the course of the disease, but also in all surgical affections which are accompanied at any stage by profuse suppuration.—*Med.-Chir. Transactions.*—*Boston Med. and Surg. Jour.*

